# Effects of Raw Material Source on the Properties of CMC Composite Films

**DOI:** 10.3390/polym14010032

**Published:** 2021-12-22

**Authors:** Yao Yao, Zhenbing Sun, Xiaobao Li, Zhengjie Tang, Xiaoping Li, Jeffrey J. Morrell, Yang Liu, Chunli Li, Zhinan Luo

**Affiliations:** 1 Yunnan Key Laboratory of Wood Adhesives and Glue Products, Southwest Forestry University, Kunming 650224, China; yaoy1012@163.com (Y.Y.); sunzhenbing66@163.com (Z.S.); lxb15925024878@163.com (X.L.); zhengjietang@163.com (Z.T.); LCL2106448925@163.com (C.L.); lzn5960929@163.com (Z.L.); 2 International Joint Research Center for Biomass Materials, Southwest Forestry University, Kunming 650224, China; 3 National Centre for Timber Durability and Design Life, University of the Sunshine Coast, Brisbane, QLD 4102, Australia; 4 Qingdao Huicheng Adhesive Co., Ltd., Qingdao 266021, China; liuyang801129@126.com

**Keywords:** Chinese pine wood, pine needles, bamboo culms, bamboo leaves, industrial hemp hurd, CMC, DS, mechanical properties, TG, XRD, FTIR

## Abstract

Sodium carboxymethyl cellulose (CMC) can be derived from a variety of cellulosic materials and is widely used in petroleum mining, construction, paper making, and packaging. CMCs can be derived from many sources with the final properties reflecting the characteristics of the original lignocellulosic matrix as well as the subsequent separation steps that affect the degree of carboxy methyl substitution on the cellulose hydroxyls. While a large percentage of CMCs is derived from wood pulp, many other plant sources may produce more attractive properties for specific applications. The effects of five plant sources on the resulting properties of CMC and CMC/sodium alginate/glycerol composite films were studied. The degree of substitution and resulting tensile strength in leaf-derived CMC was from 0.87 to 0.89 and from 15.81 to 16.35 MPa, respectively, while the degree of substitution and resulting tensile strength in wooden materials-derived CMC were from 1.08 to 1.17 and from 26.08 to 28.97 MPa, respectively. Thus, the degree of substitution and resulting tensile strength tended to be 20% lower in leaf-derived CMCs compared to those prepared from wood or bamboo. Microstructures of bamboo cellulose, bamboo CMC powder, and bamboo leaf CMC composites’ films all differed from pine-derived material, but plant source had no noticeable effect on the X-ray diffraction characteristics, Fourier transform infrared spectroscopy spectra, or pyrolysis properties of CMC or composites films. The results highlighted the potential for using plant source as a tool for varying CMC properties for specific applications.

## 1. Introduction

Sodium carboxymethyl cellulose (CMC) derived from the cellulose-containing materials is widely used in petroleum extraction, cement modification, textile production, paper making, soil improvement, and water pollution treatments [1,2,3]. While preparation of CMCs via acid-catalyzed reactions of cellulose with chloroacetic acid is relatively straightforward, the properties of the resulting product can vary widely depending on the plant source as well as the method of cellulose separation, which produces different degrees of substitution of carboxyl methyl groups on the cellulose hydroxyls. These differences have stimulated research to identify alternative sources for CMC including plant foliage and bark [4,5,6,7,8]; for example, the DS (degree of substitution) of CMC derived from corn straw was between 0.6 to 0.7 [4] and the DS of CMC derived from rice straw and reed were lower than 1.0 [5]. Foliage has the advantage of being continually harvested, while bark is often a low-value by-product of timber processing. The resulting materials can then be used to create biodegradable plastics to replace petroleum-derived materials or be combined with other materials to produce antimicrobial systems [9,10,11]. Compounded CMC, glycerol, dioscorea mucus, and Ag nanoparticles to prepare a material with strong antibacterial property and its maximum tensile strength was 12.21 MPa [10]. A composite film was prepared by adding okra mucus and nano zinc oxide in the CMC solution, and the inhibition diameters of the optimal composite sample against *Glucose aureus* and *Escherichia coli* were 14.35 ± 0.21 mm and 10.31 ± 0.21 mm, respectively, but its tensile strength was 10.26 ± 0.66 MPa [11].

Film properties can be further amended with extenders and thickeners such as starch, gelatin, pectin, and sodium alginate [2], while chitosans can be used to improve tensile strength or limit microbial attack [12,13]. The tensile strength and elongation at the break of the CMC composites prepared with 1.5% sodium alginate, 0.5% CMC, and 1.5% chitosan were 65.32 MPa and 17.85%, respectively [12]. Sodium alginate, CMC, and pyrogallic acid were used to make an antibacterial material for food packaging, and its tensile strength and elongation at the break were 24.22 ± 0.58 MPa and 39.60 ± 0.28%, respectively, and the inhibition diameters of glucose aureus and escherichia coli were 34.0 ± 1.1 mm and 18.0 ± 1.0 mm, respectively [13]. Glycerol, sorbitol, xylitol, and fructose are commonly used as plasticizers in CMC composites, but they can also enhance oxygen resistance [14,15]. Glycerol has the best plasticizing effect and the elongation at the break increases from 68.1% to 69.6% as the addition of glycerol in starch/sodium alginate/CMC composites increases from 0% to 7% [14]. Adding glycerol to sodium alginate/CMC composites can improve its oxygen resistance, and the optimum addition amount is 3% [15]. There is a diverse array of potential CMC additives, but there are relatively few studies comparing the properties of CMC films from different plant sources.

The objective of this study was to assess the impact of five different cellulose sources on the resulting properties of CMC alone or in a composite film.

## 2. Materials and Methods

### 2.1. Materials

The materials were obtained from Kunming, Yunnan Province (Table 1). All materials were air-dried and ground to powder to pass through a 40- to 60-mesh screen prior to processing. The materials differed in terms of % of lignin, hemicellulose, and cellulose with hemp hurd having the lowest lignin content and bamboo, pine wood, and hemp hurd having the most cellulose.

### 2.2. Cellulose Preparation

The ground powder of each material was soaked in an excess of 95% ethanol for 6 h at 70 °C to remove fatty acids. The 5-g samples were then collected by filtration, transferred to a 500-mL flask along with a mixture of 5 mL of glacial acetic acid, 2.5 g of sodium chlorite, and 375 mL of distilled water, and heated at 75 °C for 1 h. The solution was decanted, and 5 mL of glacial acetic acid and 2.5 g sodium chlorite were added and heated for an additional hour. The process was repeated until the material was white, indicating lignin digestion. The solids were collected by filtration and treated with 75 mL of 17.5% aqueous NaOH at room temperature for 45 min to remove the hemicelluloses. Then, the samples were neutralized by repeated washing with distilled water before being dried at 104 °C and stored in a dessicator until needed.

### 2.3. CMC and CMC Composition Films’ Preparation

**Figure 1:**
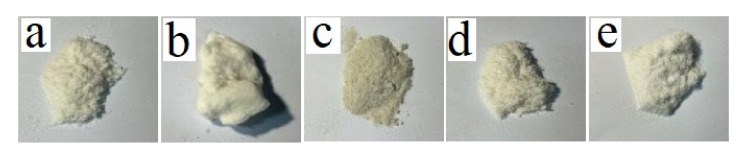
Examples of CMC powders from (**a**) pine wood, (**b**) bamboo, (**c**) pine needles, (**d**) bamboo leaves, and (**e**) hemp hurd.

CMC preparation: Four g of cellulose, 80 mL of 95% ethanol, and 20 mL of 30% NaOH solution were mixed and stirred for 60 min at 30 °C. Then, 5 g of sodium chloroacetate were added and the temperature was increased to 65 °C and held for 3 more hours with stirring. Glacial acetic acid (90%) solution was added to reduce the pH of the mixture and then the samples were washed with alcohol until the pH was 7. The neutralized samples were oven-dried at 65 °C and stored for later use (Figure 1).

**Figure 2:**
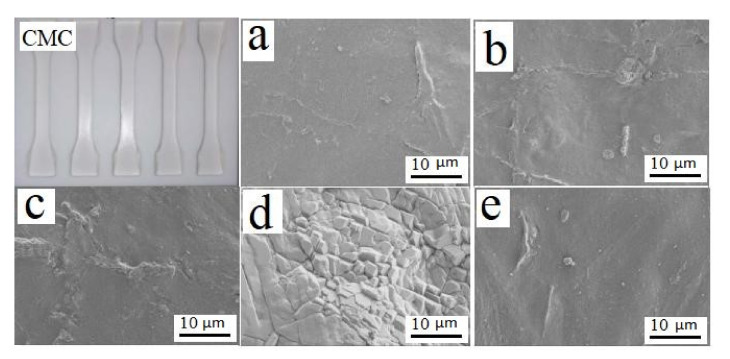
Example of CMC composite dog-bones used to assess tensile strength and scanning electron micrographs of CMC composites derived from (**a**) pine wood, (**b**) bamboo culm, (**c**) pine needles, (**d**) bamboo leaves, and (**e**) hemp hurd, showing slightly different topographies based upon parent material.

CMC film preparation: Two g of CMC powder were dispersed in 98 mL of distilled water and stirred at 900–1000 rpm at 70 °C until the CMC was completely dissolved. The solution was amended with 1.4 g of sodium alginate and 0.25 g of glycerol and thoroughly mixed before being sonicated to remove air bubbles. The resulting liquid was placed in a polytetrafluoroethylene (PTFE) mould and allowed to solidify (Figure 2).

### 2.4. Tensile Properties and Opacity of CMC Composites’ Films

Tensile strength (MPa) and elongation at break (%) were measured on 10 0.089- to 0.098-mm by 150-mm-long dog-bone samples of each material on a Universal Testing Machine according to procedures described in GB/T 1040.1-2006 (Plastics Determination of tensile properties). A load was applied to failure at a rate of 1 mm/min.

The opacity of the CMC composite films was tested by cutting 10- by 40-mm-long samples and placing them on the inner surface on one side of a cuvette and then measuring absorbance at 600 nm on a 752^#^ ultraviolet spectrophotometer (XP-Spectrum Company, Shanghai, China). Five tests were performed for each material.

### 2.5. Material Charaterization

Microstructure: Samples of the parent materials, the extracted cellulose, reacted CMC powder, and the resulting CMC composite film were placed on an aluminium grid and examined by field emission scanning electron microscopy on a Nova NanoSEM 450 microscope (FEI, Hillsboro, OR, USA). A minimum of five fields were examined per material.

Degree of CMC Substitution: The degree of substitution on the hydroxyls had important effects on the resulting CMC properties. The degree of substitution was determined by the ash alkali method [4], wherein 1.5 g of CMC powder were placed into a crucible and washed four to five times with 80% ethanol at 50 °C to 70 °C to remove any residual soluble salt and then washed once with 100% ethanol. The samples were dried at 104 °C. Then, 1 g of the oven-dried sample was placed in a crucible and then heated in a muffle furnace of 300 °C until no smoke was observed. The temperature was then increased to 700 °C for 15 min. The sample was removed from the oven and allowed to cool to 200 °C before being transferred to a beaker with 100 mL of distilled water and 50 mL of 0.1 mol/L sulfuric acid standard titration solution. The beaker was heated to boiling for 10 min. Then 2–3 drops of methyl red indicator solution were added. A 0.1 mol/L sodium hydroxide standard solution was added dropwise until the solution turned from red to white. The amount required to reach the end point was then used to calculate the degree of substitution (*DS*) based on Equations (1) and (2), as follows.

(1) $$B=\frac{c_{1}V_{1}-c_{2}V_{2}}{m}$$

(2) $$DS=\frac{162B}{1000-80B}=\frac{0.162B}{1-0.08B}$$

where B is the Amount of carboxymethyl substance contained in the sample, mmol/g; m is the Quality of the sample, g; $c_{1}$ is the Concentration of sulfuric acid standard titration solution, mol/L; $V_{1}$ is the Volume of sulfuric acid standard titration solution, mL; $c_{2}$ is the Concentration of sodium hydroxide standard titration solution, mol/L; and $V_{2}$ is the Volume of sodium hydroxide standard titration solution, mL.

X-ray Diffractometer analysis: The relative degree of crystallinity of the raw materials, the extracted cellulose, and the reacted CMC was examined by X-ray diffractometry on a Rigaku Ultima IV X-ray diffractometer (Rigaku Corp, Tokyo, Japan) (XRD, Ulti,) using a scanning angle from 5° to 80°, a step size of 0.026° (accelerating current = 30 mA and voltage = 40 kV), and Cu-Kα radiation of ℷ = 0.154 nm.

Fourier Transform Infrared Spectroscopy (FTIR): Extracted cellulose, CMC, and CMC composite powder from each plant source were mixed with KBr, pressed into a pellet, and analyzed on a Nicolet i50 FTIR Analyzer (Thermo Scientific, Waltham, MA, USA). Samples were subjected to 64 scans and the resulting spectra were baseline corrected and then analyzed for differences in spectra for different raw materials.

Thermogravimetric (TG) analysis: Approximately 5.0 to 6.0 mg of the original air-dried raw materials as well as the extracted cellulose, the CMC, and the CMC composites film were ground to pass an 80-mesh to 120-mesh and placed into sample holders for analysis on a TGA92 thermo gravimetric analyzer (KEP Technologies EMEA, Caluire, France). N_2_ was used as the shielding gas and Al_2_O_3_ as the reference compound. The temperature was increased from room temperature (approx. 20–23 °C) to 800 °C at a rate of 10 °C/min to produce thermogravimetric curves.

## 3. Results and Discussion

### 3.1. The Physical and Mechanical Properties of CMC Composites’ Films

**Figure 3:**
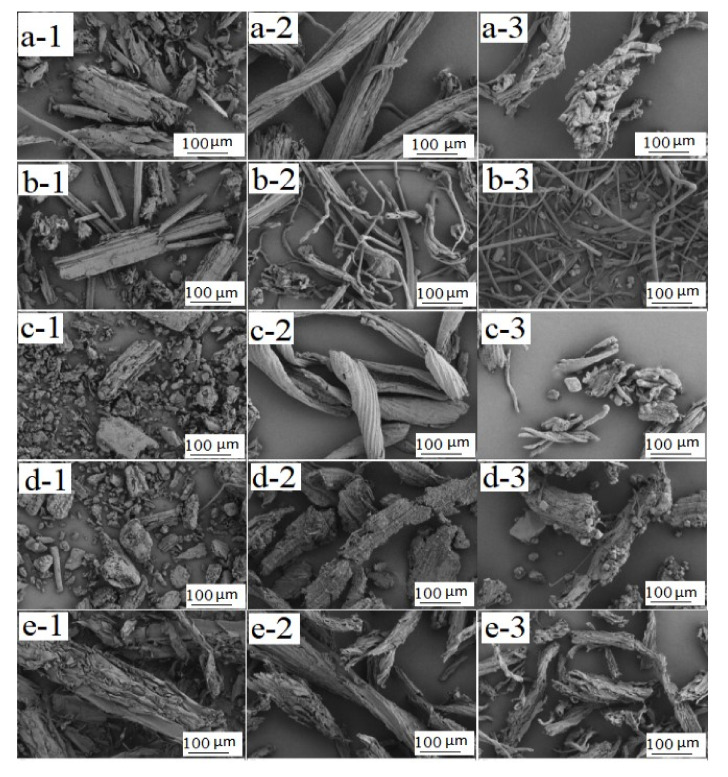
SEM images showing microstructure differences for the (**1**) the raw material, (**2**) the extracted cellulose, and (**3**) the resulting CMC derived from (**a**) pine wood, (**b**) bamboo culm, (**c**) pine needles, (**d**) bamboo leaves, and (**e**) hemp hurd.

The Color of the CMC powders varied slightly with source and the resulting films contained small particles or protrusion (Figure 1 and Figure 2). CMCs prepared from bamboo leaves contained slender particles, which were not present in the other materials (Figure 3).

The degrees of substitution of hydroxyls were similar for CMCs from bamboo foliage and pine needles but were nearly 20% lower than those for CMCs from pine wood, bamboo culm, or hemp hurd (Table 2). The lower levels of substitution on leaves or needles were surprising since the cellulose in these materials should be less heavily lignified and, therefore, more accessible to substitution. Tensile strength was also lower for the foliage-derived CMCs. The tensile strength of CMC composite films ranged from 15.81 MPa to 28.97 MPa, which were much higher than results from previous studies [9,10,11,13], while elongation at break (%) ranged from 3.37% to 6.60%, which were lower than previous reports [12]. Chitosan addition might help improve tensile strength [12] and will be explored in future studies.

Opacity of the resulting films was similar for the pine wood, bamboo culm, and foliage, while films from pine needles were slightly higher.

Increased degree of substitution was highly correlated with increased tensile strength (r = 0.94), which is in line with previous research [7]. Elongation at break and opacity were both negatively correlated with degree of substitution (r = −0.68 and −0.49, respectively), suggesting that increased substitution disrupted the integrity of the cellulose chain. CMCs can be classified by degree of substitution as low (0.4 to 1.0), high (1.0 to 1.6), or super high (1.7 to 3.0), respectively [5,8,9]. The foliage-derived CMCs had low degrees of substitution and could be used for petroleum extraction or cement modification. CMCs made from pine wood, bamboo culm, or hemp hurd had high degrees of substitution and could be used for making food package [5]. The results illustrated the potential for obtaining CMCs from specific plant materials based upon ultimate application.

**Figure 4:**
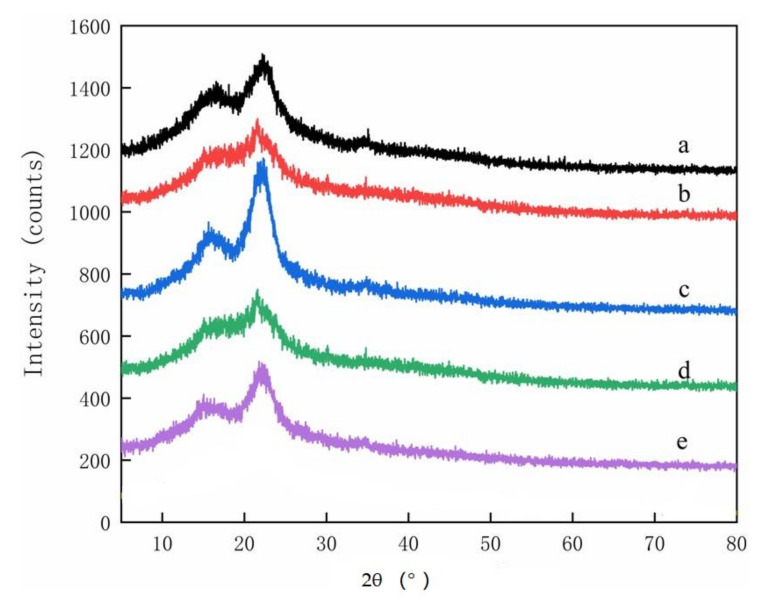
XRD spectra of parent materials used to produce CMCs: (**a**) pine wood, (**b**) bamboo culm, (**c**) pine needles, (**d**), bamboo leaves, or (**e**) hemp hurd.

**Figure 5:**
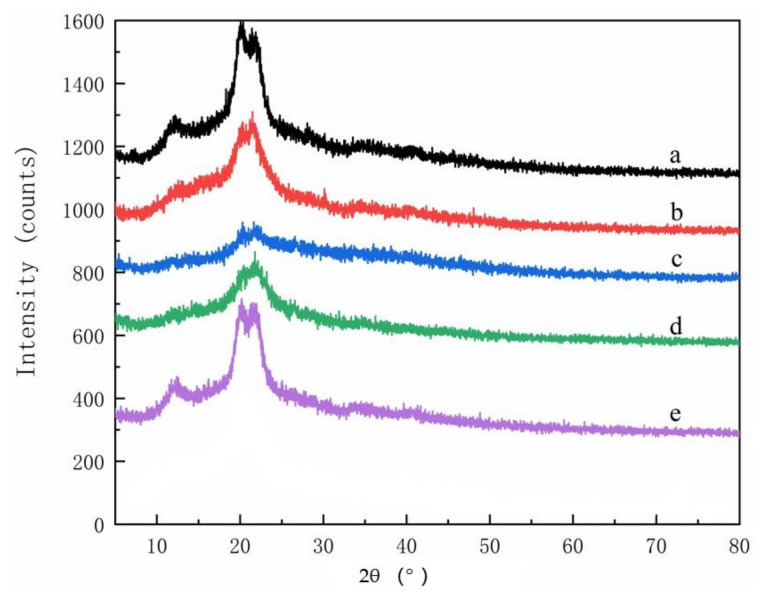
XRD spectra of cellulose derived from (**a**) pine wood, (**b**) bamboo culm, (**c**) pine needles, (**d**) bamboo leaves, or (**e**) hemp hurd.

**Figure 6:**
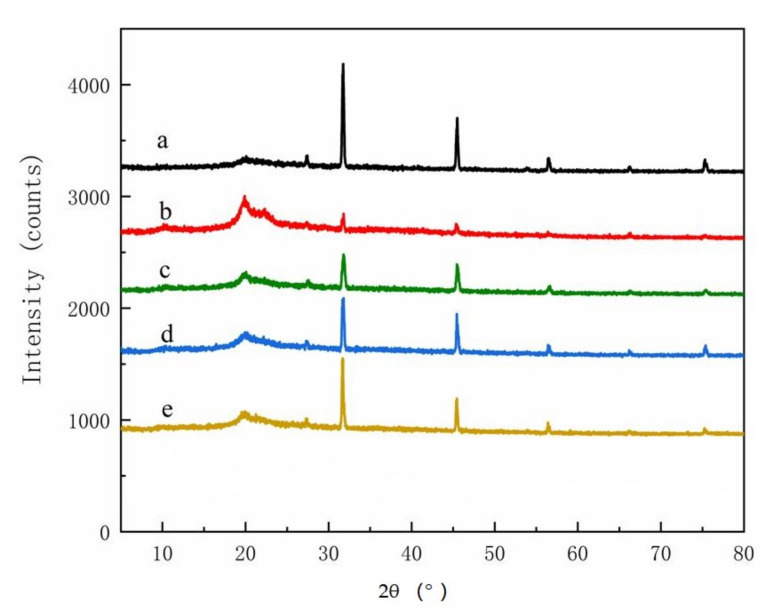
XRD spectra of CMCs derived from (**a**) pine wood, (**b**) bamboo culm, (**c**) pine needles, (**d**) bamboo leaves, or (**e**) hemp hurd.

XRD spectra from the parent materials contained two featured cellulose peaks with 2θ = 15.8° and 2θ = 20.8°, respectively (Figure 4). The cellulose peak at 2θ = 15.8° decreased in the isolated cellulose (Figure 5), although it was still present. The parent materials or the extracted cellulose all contained Type I cellulose, which is one of the six cellulose isomers [20,21]. No peaks were present at 2θ = 15.8° for any material after extraction and reaction with chloroacetic acid, indicating successful conversion into CMC [22], but four new peaks were observed between 32° to 75° (Figure 6). These peaks have not been noted in previous studies and merit further study.

### 3.2. The FTIR Characterization of CMC Composites

**Figure 7:**
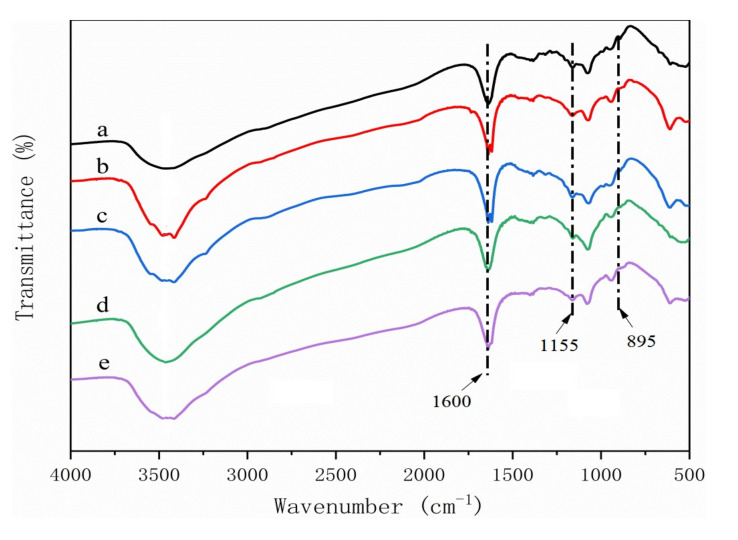
FTIR spectra of cellulose derived from (**a**) pine wood, (**b**) bamboo culm, (**c**) pine needles, (**d**) bamboo leaves, or (**e**) hemp hurd.

**Figure 8:**
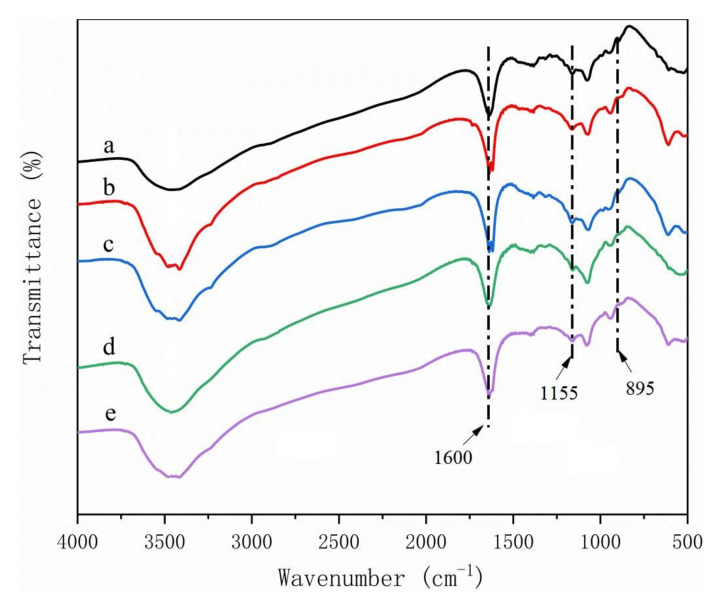
FTIR spectra of CMCs derived from (**a**) pine wood, (**b**) bamboo culm, (**c**) pine needles, (**d**) bamboo leaves, or (**e**) hemp hurd.

**Figure 9:**
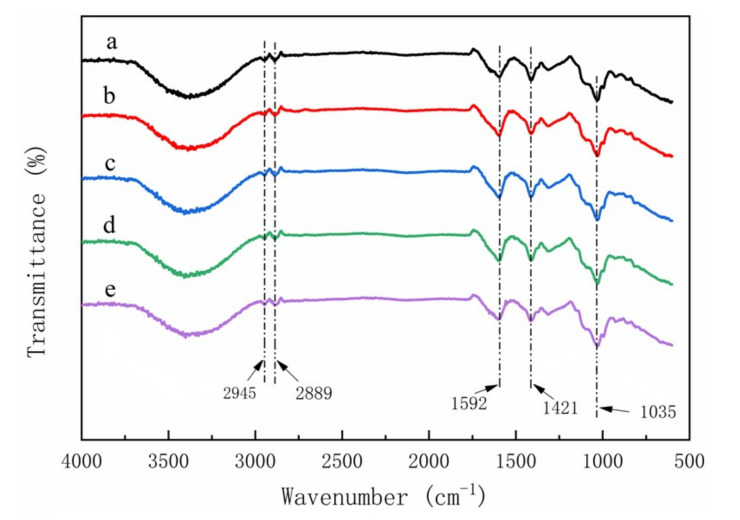
FTIR spectra of CMC composite films derived from (**a**) pine wood, (**b**) bamboo culm, (**c**) pine needles, (**d**) bamboo leaves, or (**e**) hemp hurd.

Peaks typically found for materials containing lignin and hemicellulose were absent form the cellulose regardless of raw material source [23,24] (Figure 7). These results indicated that the extraction process successfully removed the polymers, leaving a pure cellulose residue for CMC production. Two peaks at 1592 cm^−1^ and 1421 cm^−1^ corresponding to COO^—^ stretching of the carboxylic group and O-H stretching of CMC, respectively, were found in CMC for all five materials, indicating that the materials were successfully reacted (Figure 8) [5,25]. These same peaks were also detected in spectra from the CMC composite films, suggesting that reactions did not alter the hydroxyl interactions. However, three new peaks were observed at 1035 cm^−1^, 2889 cm^−1^, and 2945 cm^−1^ in the composites (Figure 9), which means that the CMC composites were different from the CMC powder and this new peak need be researched more in the future.

### 3.3. The Pyrolysis Characteristics of CMC Composites

**Figure 10:**
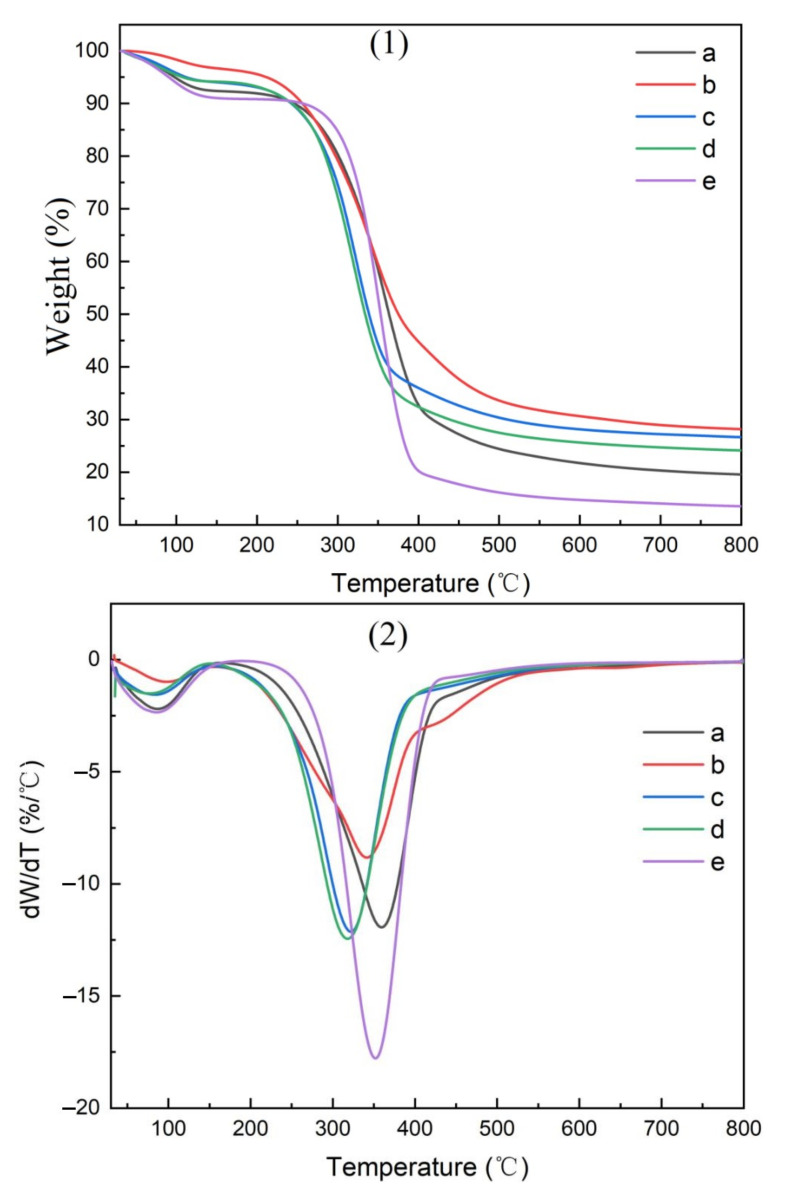
TG curves of materials used to produce CMCs derived from (a) pine wood, (b) bamboo culm, (c) pine needles, (d), bamboo leaves, or (e) hemp hurd, shown as thermogravimetric analysis (**1**) or differential thermogravimetric analysis (**2**).

**Figure 11:**
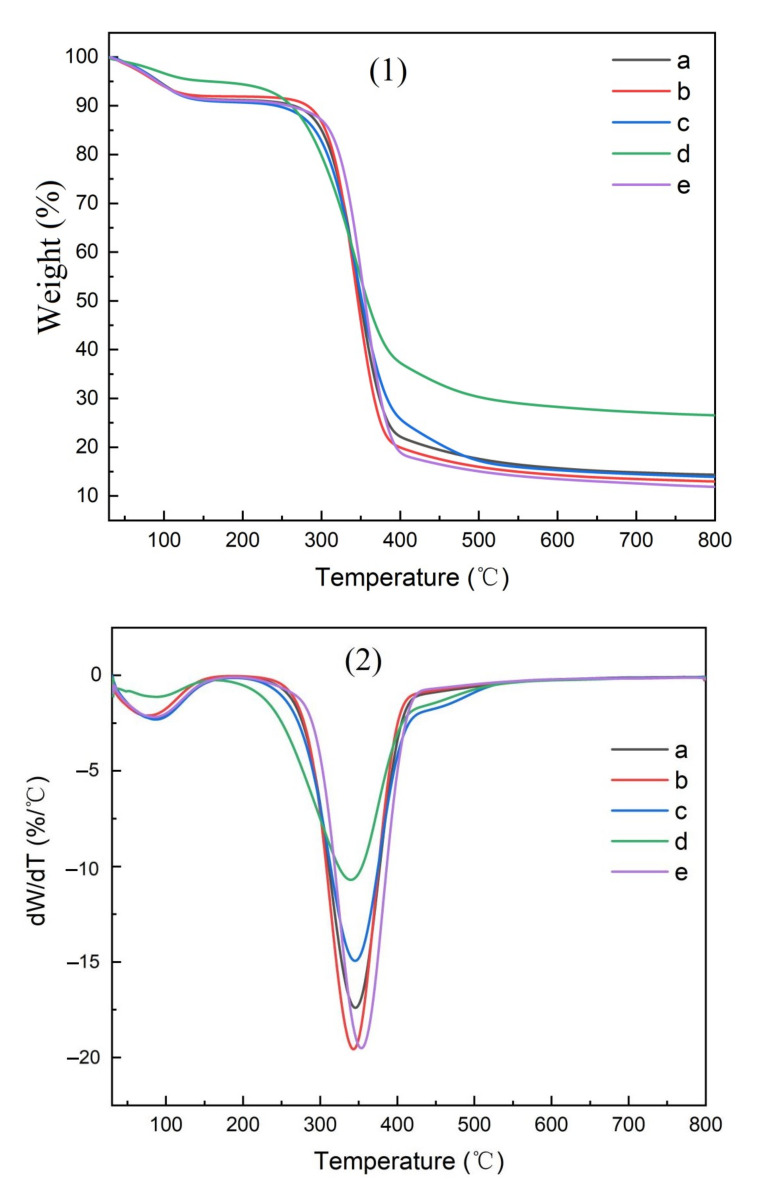
TG curves of cellulose derived from (a) pine wood, (b) bamboo culm, (c) pine needles, (d), bamboo leaves, or (e) hemp hurd, shown as thermogravimetric analysis (**1**) or differential thermogravimetric analysis (**2**).

**Figure 12:**
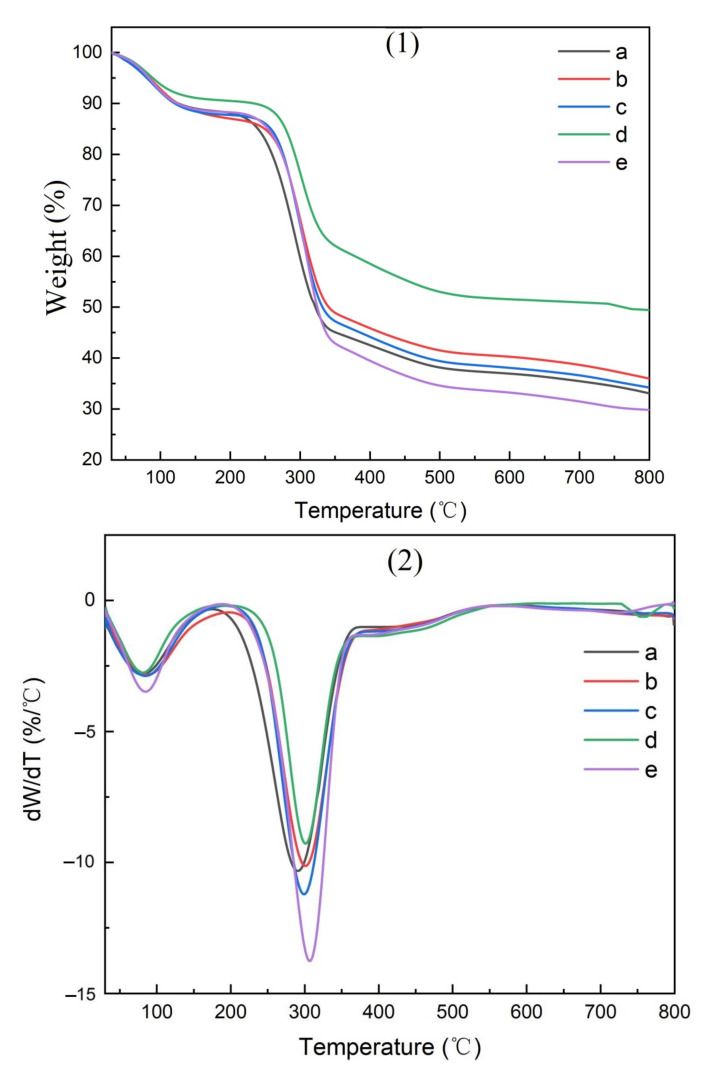
TG curves of CMCs derived from (a) pine wood, (b) bamboo culm, (c) pine needles, (d), bamboo leaves, or (e) hemp hurd, shown as thermogravimetric analysis (**1**) or differential thermogravimetric analysis (**2**).

**Figure 13:**
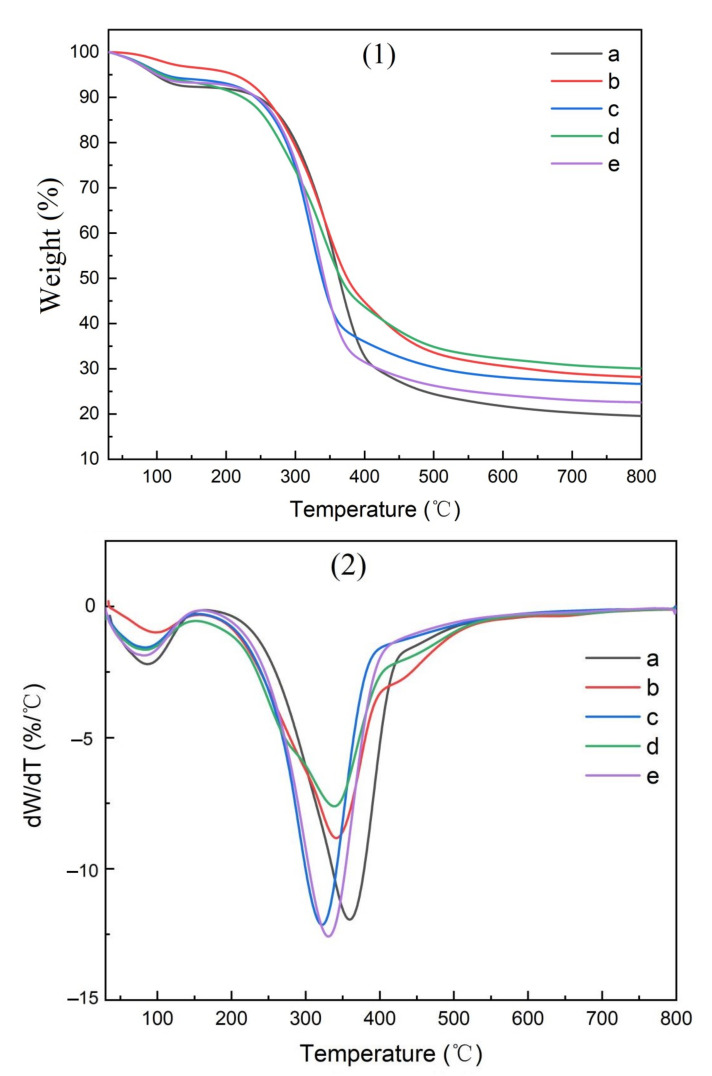
TG curves of CMC-composite films derived from (a) pine wood, (b) bamboo culm, (c) pine needles, (d), bamboo leaves, or (e) hemp hurd, shown as thermogravimetric analysis (**1**) or differential thermogravimetric analysis (**2**).

The thermogravimetric (TG) curves for the parent materials, isolated cellulose, CMC, and CMC composite films are shown in Figure 10, Figure 11, Figure 12 and Figure 13, respectively.

The amount of residue remaining after pyrolysis varied widely between raw materials, reflecting the inorganic elements present (Table 3). The highest residues in parent materials were found with bamboo culms and leaves as well as pine needles, while residues were lower in pine wood and hemp hurd. Bamboo contained elevated levels of silica, while pine timber contained a number of minerals. Bamboo leaves were associated with the highest residues in purified cellulose and the resulting CMC, while hemp hurd was associated with the lowest residue levels for both of these materials.

Two pyrolysis peaks were noted in the resulting TG curves (Figure 10, Figure 11, Figure 12 and Figure 13), supporting previous studies [25]. The first peak occurred near 100 °C, reflecting the removal of residual moisture. The second pyrolysis peak for pine, bamboo, pine needles, bamboo leaves, and hemp hurd parent materials occurred between 315 and 360 °C (Table 4), while the peak occurred between 340 and 352 °C for the extracted cellulose and 290 and 310 °C for the CMC. The peak occurred between 320 and 360 °C for the CMC film. The wider range in peak temperatures for the parent materials was consistent with their varying levels of cell wall polymers. Cellulose extraction followed by CMC production resulted in more uniform peak temperatures, while film formation resulted in an increase in the peak temperature and more variability among parent materials. The results, however, were fairly similar and suggest that the parent materials produced uniform cellulose, CMC, and CMC film derivatives.

## 4. Conclusions

Raw material source had marked effects on the degree of substitution of CMCs and, ultimately, the tensile strength of the resulting CMC films. The results highlight the potential for creating CMCs for selective applications by careful selection of parent materials. Leaves appear to be better suited for fillers for petroleum extraction or cement, while wood tissues from hemp hurd, bamboo, and pine were more suited for creating films for packing. Further studies are planned with chitosan as an additive.

## Figures and Tables

**Table 1:** Lignin, cellulose, and hemicelluloses content of materials used to produce CMCs.

Source	Lignin (%)	Holo-Cellulose (%)	Cellulose (%)	Hemicellulose (%)	Source
Pine wood	23.0	-	39.9	14.9	[16]
Bamboo	26.4 (0.04)	66.7 (0.02)	43.9 (0.02)	22.8 (0.03)	[17]
Pine needles	29.3 (0.3)	40.8 (0.3)	20.5 (0.07)	20.3 (0.4)	[18]
Bamboo leaves	25.2 (0.8)	57.3 (0.5)	19.5 (0.4)	37.7 (0.5)	[19]
Hemp Hurd	20.9 (0.1)	70.8 (0.1)	42.9 (0.1)	27.9 (0.2)	[19]

**Table 2:** Characteristics of CMCs and CMC films produced from different cellulosic sources.

Source	Degree of Substitution	Tensile Strength (MPa)	Elongation at Break (%)	Opacity (A/mm)
Pine wood	1.17 (0.026)	27.50 (1.93)	3.50 (1.23)	4.34 (0.27)
Bamboo culm	1.08 (0.046)	28.97 (3.17)	3.50 (1.42)	3.67 (0.26)
Pine needles	0.87 (0.025)	15.81 (2.19)	6.60 (0.79)	6.60 (0.18)
Bamboo leaves	0.89 (0.071)	16.35 (1.27)	3.67 (0.34)	4.34 (0.31)
Hemp hurd	1.12 (0.088)	26.08 (2.69)	3.37 (0.39)	5.22 (0.14)

Values represents means of three samples for degree of substitution, 10 samples for tensile strength and elongation, and five samples for opacity, while figures in parentheses represent one standard deviation.

**Table 3:** Residue remaining (as a % of the original material) after pyrolysis.

Materials	Residual Material (%)				
	Pine Wood	Pine Needle	Bamboo Culm	Bamboo Leaves	Hemp Hurd
Parent material	19.50	26.42	27.82	25.12	14.32
Cellulose	14.46	14.12	12.98	26.66	11.84
CMC	33.01	33.92	35.84	48.08	26.60
CMC-composite film	19.72	26.20	27.82	29.44	22.42

**Table 4:** Temperature at which the second peak was observed during pyrolysis.

Materials	Peak Temperature (°C)				
	Pine Wood	Pine Needle	Bamboo Culm	Bamboo Leaves	Hemp Hurd
Parent materials	360	320	340	314	351
Cellulose	342	342	340	342	352
CMC	290	298	298	290	310
CMC-composite film	360	320	340	340	325

## Data Availability

The data presented in this study are available from the listed authors.
